# Genetic Population Structure of the Coral Reef Sea Star *Linckia laevigata* in the Western Indian Ocean and Indo-West Pacific

**DOI:** 10.1371/journal.pone.0165552

**Published:** 2016-10-31

**Authors:** Levy Michael Otwoma, Marc Kochzius

**Affiliations:** 1 Kenya Marine and Fisheries Research Institute, P.O. BOX 81651, Mombasa, Kenya; 2 Marine Biology, Vrije Universiteit Brussel, Pleinlaan 2, 1050, Brussels, Belgium; National Cheng Kung University, TAIWAN

## Abstract

The coral reef sea star *Linckia laevigata* is common on shallow water coral reefs of the Indo-West Pacific. Its large geographic distribution and comprehensive data from previous studies makes it suitable to examine genetic differentiation and connectivity over large geographical scales. Based on partial sequences of the mitochondrial cytochrome oxidase I (COI) gene this study investigates the genetic population structure and connectivity of *L*. *laevigata* in the Western Indian Ocean (WIO) and compares it to previous studies in the Indo-Malay-Philippines Archipelago (IMPA). A total of 138 samples were collected from nine locations in the WIO. AMOVA revealed a low but significant Φ_ST_-value of 0.024 for the WIO populations. In the hierarchical AMOVA, the following grouping rejected the hypothesis of panmixia: (1) Kenya (Watamu, Mombasa, Diani) and Tanzanian Island populations (Misali and Jambiani) and (2) the rest of the WIO sites (mainland Tanzania and Madagascar; Φ_CT_ = 0.03). The genetic population structure was stronger and more significant (Φ_ST_ = 0.13) in the comparative analysis of WIO and IMPA populations. Three clades were identified in the haplotype network. The strong genetic differentiation (Φ_CT_ = 0.199, P < 0.001) suggests that Indo-West Pacific populations of *L*. *laevigata* can be grouped into four biogeographic regions: (1) WIO (2) Eastern Indian Ocean (3) IMPA and (4) Western Pacific. The findings of this study support the existence of a genetic break in the Indo-West Pacific consistent with the effect of lowered sea level during the Pleistocene, which limited gene flow between the Pacific and Indian Ocean.

## Introduction

The tropical Indo-West Pacific region hosts the world’s greatest diversity of marine shallow water species [1], with species richness decreasing longitudinal and latitudinal from the centre [2–4]. Besides harbouring the world’s richest marine shallow water biodiversity, this region experienced complex geologic events that strongly influenced distribution patterns of marine taxa. In particular, the frequent global fluctuation of sea level by up to 120 m created a vicariant barrier, which has been principally hypothesised to have caused genetic partitioning between the Pacific and Indian Ocean in numerous taxa [5]. However, findings from molecular genetic studies on species spanning the Indo-West Pacific show discordant population structures, implying that processes influencing the genetic structure of taxa, and hence evolution in the region, are not uniform across species. For instance, results on anemonefish *Amphiprion* spp [6], lionfishes *Pterois* spp [7], butterflyfishes *Chaetodon* spp [8], and sea stars *Linckia* spp [9] indicate a phylogenetic break between the Indian and Pacific Ocean, supporting the notion of allopatric speciation in separated ocean basins. On the contrary, organisms such as the sea urchins *Eucidaris*, *Diadema*, and *Tripneustes* [10–12], the marine snails *Echinolittorina reticulate* [13] and *Thyca crystallina* [14] lack this phylogeographic break across the Indo-Malay-Philippines Archipelago (IMPA). The lack of differentiation is attributed to their ability to have sustained dispersal throughout the IMPA during the glacial maxima, swiftly re-established gene flow after glacial maxima, or loss of their divergent lineages [15].

Most marine shallow water species are sedentary in nature, depending on their planktonic larval stage for long distance dispersal. Usually, the newly spawned larvae drift with the ocean currents, travelling 1000s of kilometres. This aspect makes larval dispersal in marine organisms a crucial factor, influencing population dynamics, population persistence, and range expansion. Genetic markers provide a tool that can determine the extent of larval dispersal. Generally, successfully dispersed larvae will leave a genetic trail of their movements, providing a proxy of estimating realised gene flow or connectivity [16]. Species with longer pelagic larval duration (PLD) are expected to exhibit higher gene flow and connectivity than those possessing a shorter PLD [17]. For example, it has been shown that broadcasting acroporid corals with a long PLD have higher gene flow than brooding corals of the genera *Stylophora* and *Seriatopora*, which have a shorter PLD [18]. Although numerous studies support the simple correlation of realised dispersal to traits affecting dispersal, such as PLD, findings in widespread organisms, such as the mantis shrimp *Haptosquilla pulchella* and the tropical abalone *Haliotis asinina* contradict this pattern. *Haptosquilla pulchella*, with a long-distance dispersal capacity, shows strong genetic structure among Indo-West Pacific populations, while *Haliotis asinina* displays high levels of gene flow, despite having a restricted PLD [19, 17]. The existing discrepancy between gene flow and PLD proves that gene flow or connectivity among marine populations is not only affected by larval behaviour, but also by ocean currents [20], topographic features, local adaptation [17], geographical distance [21], and interactions with other species [19].

The blue sea star *Linckia laevigata* is a common benthic organism on Indo-West Pacific coral reefs, with a wide distribution from the Western Indian Ocean (WIO) to southeastern Polynesia [22–23]. It abundantly occurs on the shallow reef flat or coral patches in the lagoons of fringing reefs [23]. *Linckia laevigata*, such as other asteroids, is a sedentary organism depending on its planktonic larval stage for long distance dispersal. Due to its wide distribution and comprehensive data from previous studies, it is a suitable species to examine and understand levels of connectivity in coral reef ecosystems in the Indo-West Pacific [24]. It reproduces sexually, with an external fertilisation that involves the fusion of gametes freely in the water column. The peak breeding period occurs during the summer months, such as May to August in the case of the population in Guam [23] and may vary regionally. Metamorphosis takes at least 22 days in the larvae of *L*. *laevigata* [25], suggesting that its PLD may be longer than 3 weeks.

**Fig 1:**
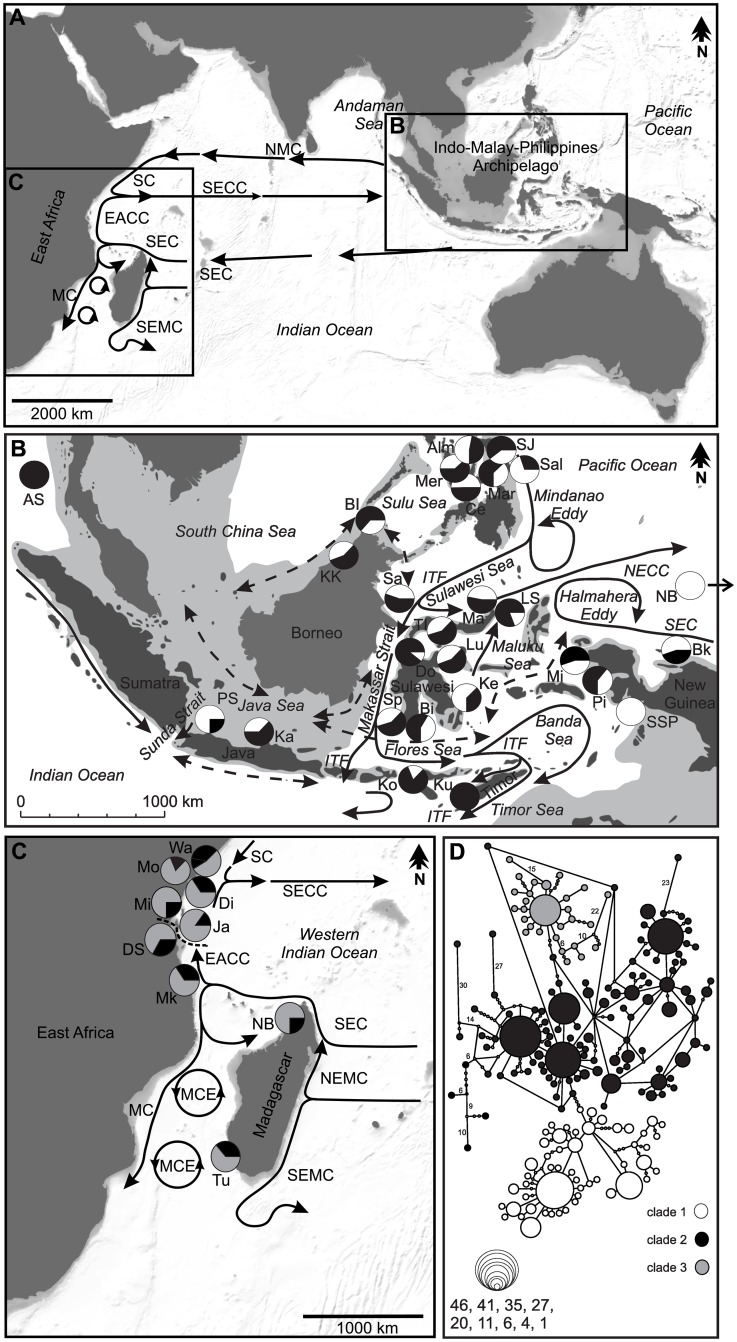
Maps of the (A) Indo-West Pacific, (B) Indo-Malay-Philippines Archipelago (IMPA) and (C) Western Indian Ocean (WIO) with sample sites (for abbreviations see Tables 1 and 2), as well as oceanographic patterns (WIO; during the Northeast Monsoon), with dominant (solid lines) and seasonally changing (dashed lines; IMPA) currents [28, 29, 30, 31, 32]. EACC, East African Coast Current; ITF, Indonesian Throughflow; MC, Mozambique Current; MCE, Mozambique Channel Eddies; NEMC, Northeast Madagascar Current; NECC, Northern Equatorial Counter Current; SC, Somali Current; SEC, Southern Equatorial Current; SECC, South Equatorial Counter Current; SEMC, Southeast Madagascar Current. The dashed line in (C) indicates the genetic differentiation between northern and southern populations in the WIO. Pleistocene maximum sea level low stand of 120 m is indicated by the light grey area ([5] IMPA). Pie charts represent the proportion of clades defined in the haplotype network at the different sample sites. (D) Haplotype network based on partial mitochondrial cytochrome oxidase I (COI) sequences. Large circles represent haplotypes and lines represent one mutational step. Numbers indicate additional mutational steps, while small circles represent missing intermediate haplotypes. The size of the circles is proportional to haplotype frequency. (A) and (C) are based on maps drawn with the software MapCreator, (B) is based on a map from [5].

The WIO is a constituent of the tropical Indo-West Pacific, representing an important biogeographic region of tropical seas [26]. Despite being amongst the most biologically diverse tropical ecosystems, the genetic structure of WIO coral reef species remains one of the least studied globally [27]. For that reason, most coral reef species in the WIO are still managed without any consideration that some populations might be restricted in larval exchange. The WIO has a complex current system that can facilitate long-distance larval dispersal and can form barriers. The South Equatorial Current (SEC), which flows westward across the Indian Ocean, can facilitate long distance dispersal. However, the SEC bifurcates at the East African coast, forming the northward East African Coast Current (EACC) and the southward Mozambique Current, as well as the Mozambique Channel Eddies. This potentially forms an oceanographic barrier for dispersal between the northern and southern East African coast. The EACC joins the Somali Current in the North of the East African coast, forming the seasonal South Equatorial Counter Current (SECC) (Fig 1).

Although several studies have been performed on *Linckia laevigata* across the Indo-Pacific [9, 21, 24, 33], none has studied gene flow within the WIO. Therefore, to complement previous work on connectivity in *Linckia laevigata*, the present study investigates the genetic population structure, genetic diversity and historical demography of *L*. *laevigata* at nine sites in the WIO. Due to the long PLD of *Linckia laevigata* broad-scale genetic homogeneity in the WIO would be expected. Additionally, this study aims to assess connectivity of populations in the WIO to the IMPA. The comparative genetic analysis of *L*. *laevigata* populations from the WIO and IMPA complements previous research in testing the vicariance hypothesis [33], but with extensive sampling that includes multiple locations in the WIO and IMPA (Fig 1). This extensive sampling facilitates detailed, fine-scale analysis of the genetic differentiation in *Linckia laevigata* across the Indo-West Pacific. The sequence data of populations from the IMPA were obtained from two previous studies [14, 34].

## Materials and Methods

### Ethics statement

Permission to collect samples was provided by Kenya Wildlife Services, Tanzania Commission for Science and Technology (COSTEC), Zanzibar Research Committee, the University of Tuléar (Madagascar), and Offices of the Municipal Agriculturist (Marabut, Western Samar; Merida, Leyte; Catbalogan, Western Samar; Philippines). Sampling in Indonesia was permitted under the governmental agreement between the German Federal Ministry of Education and Research (BMBF) and the Indonesian Ministry for Research and Technology (RISTEK), Indonesian Institute of Sciences (LIPI), Indonesian Ministry of Maritime Affairs and Fisheries (DKP), and Agency for the Assessment and Application of Technology (BPPT). It was carried out in co-operation with Hassanuddin University (UNHAS, Makassar), Agricultural University Bogor (IPB, Bogor), and Jenderal Soedirman University (UNSOED, Purwokerto).

### Study sites and sampling

Tissue samples of *L*. *laevigata* were collected between February 2011 and February 2012 at nine sites located in the WIO (Fig 1C and Table 1). In total, 138 tissue samples were collected while SCUBA diving by cutting off a small piece of tissue (~ 1cm) from adult *L*. *laevigata* specimens without killing the animals. The tissue samples were preserved in absolute ethanol and stored at 4°C prior to DNA extraction. The analysis was restricted to blue colour morph individuals, because there is a congruent pattern between genetic and colour variation [33].

**Table 1:** Sample site, code, region and the number of individuals collected (n).

Sample site	Code	Region	Latitude (S)	Longitude (E)	n
Watamu	Wa	WIO	03°21'59”	40°00'45”	15
Mombasa	Mo	WIO	04°01'18”	39°44'24”	16
Diani	Di	WIO	04°21'03”	39°34'48”	12
Misali, West Coast of Pemba	Mi	WIO	05°16'03”	39°36'38”	6
Jambiani, East Coast of Zanzibar	Ja	WIO	06°18'45”	39°33'36”	17
Dar es Salaam	DS	WIO	06°46'13”	39°26'16”	27
Mikindani	Mk	WIO	10°15'53”	40°07'30”	10
Nosy Bé	NB	WIO	13°14'56”	47°54'43”	20
Tuléar	Tu	WIO	20°39'25”	43°46'30”	15

### DNA extraction, amplification, and sequencing

Extraction of genomic DNA was done with the Nucleospin extraction kit, following the manufacturer’s protocol. The amplification of the partial COI gene was through polymerase chain reaction (PCR), using the primers described by [35]: LC01490 (5’-GGT CAA CAA ATC ATA AAG ATA TTG G-3’) and HC02198 (5’-TAA ACT TCA GGG TGA CCA AAA AAT CA-3’). PCR was conducted in a 50 μl reaction volume containing 4 μl DNA template, 10 mM Tris-HCl (pH 9), 50 mM KCl, 0.2 mM dNTPs, 0.4 μl BSA (10 mg/ml), 0.2 μl Taq polymerase (5u/μl), 0.4 μM of each primer and 2.0–2.5 mM MgCl_2_. The following temperature profile was used to conduct PCR: 94°C for 5 min, followed by 35 cycles of 1 min at 94°C, 1.5 min at 45°C and 1 min at 72°C. Final extension was conducted at 72°C for 5 min [14]. Sequencing was done using the DyeDeoxy terminator chemistry (PE Biosystem) and an automated sequencer (ABI PRISM 310 and 3100, Applied Biosystems).

The 138 sequences analysed for this study are available in GenBank under accession numbers KF527577-KF527714. In total, 396 sequences from the IMPA were utilised for the comparative analysis (Table 2).

**Table 2:** Sample sites, number of sequences (n) and number of haplotypes (N_hp_) for *Linckia laevigata* in the Indo-Malay-Philippine Archipelago (IMPA).

Sample site	Code	Region	Source	n	Nhp
Almagro, Samar, Visayas	Alm	IMPA	[34]	19	16
Andaman Sea	AS	EIO	[14]	3	3
Bira, Sulawesi	Bi	IMPA	[14]	13	9
Banggi Islands, Borneo	BI	IMPA	[14]	15	10
Biak, New Guinea	Bk	WP	[14]	9	9
Cebu, Visayas	Ce	IMPA	[14]	10	7
Donggala, Sulawesi	Do	IMPA	[14]	10	5
Karimunjava, Java	Ka	IMPA	[14]	10	9
Kendari, Sulawesi	Ke	IMPA	[14]	11	8
Kota Kinabalu, Borneo	KK	IMPA	[14]	6	6
Komodo	Ko	IMPA	[14]	9	8
Kupang, Timor	Ku	EIO	[14]	8	8
Lembeh Strait, Sulawesi	LS	IMPA	[14]	12	9
Luwuk, Sulawesi	Lu	IMPA	[14]	9	7
Manado, Sulawesi	Ma	IMPA	[14]	13	10
Marabut, Samar, Visayas	Mar	IMPA	[34]	28	21
Merida, Leyte, Visayas	Mer	IMPA	[34]	16	14
Misool, Moluccas	Mi	IMPA	[14]	16	12
New Britain, New Guinea	NB	WP	[14]	2	2
Pisang, New Guinea	Pi	WP	[14]	13	11
Pulau Seribu, Java	PS	IMPA	[14]	9	8
Salcedo, Samar, Visayas	Sal	IMPA	[34]	25	16
Sangalaki, Borneo	Sa	IMPA	[14]	11	9
San Jose, Samar, Visayas	SJ	IMPA	[34]	36	25
Spermonde, Sulawesi	Sp	IMPA	[14]	52	28
Sebakor/Sanggala/Papisol, New Guinea	SSP	IMPA	[14]	11	8
Togian Island, Sulawesi	TI	IMPA	[14]	20	14

EIO: Eastern Indian Ocean; WP: Western Pacific.

### Genetic diversity

The software ChromasPro (version 1.5; Technelysium) was used for editing the WIO sequences. The correct species identity of each sequence was verified using the Basic Local Alignment Search Tool (BLAST) at GenBank. To ensure that only functional mitochondrial DNA was used and not nuclear pseudogenes, the sequences were translated into amino acids with the software Squint Alignment Editor (version 1.02), in order to verify that the DNA sequences code for a functional protein. The online service of FaBox (version 1.4) was used to collapse the sequences into haplotypes. A multiple sequence alignment was obtained with CLUSTAL W [36] as implemented in the software BIOEDIT (version 7.0.4.1) [37]. Haplotype diversity (h) [38] and nucleotide diversity (π) [39] were calculated with the program Arlequin (http://cmpg.unibe.ch/software/arlequin35, version 3.5.1.3) [40].

### Historical demography

Tajima’s *D*-test [41] and Fu’*Fs*-test [42] were used to test the null hypothesis of neutral evolution of the marker in the WIO sequences. Negative Tajima’s *D* values indicate selective sweeps or population expansion after a recent bottleneck event [41]. To analyse the historical demography, the mismatch distribution [43] of the sums of squares deviation (SSD) [44] and Harpending’s raggedness index (HRI) were used, which allowed for testing of the model of sudden population expansion [45]. The mismatch distribution is multimodal in populations under a demographic equilibrium and unimodal if a recent and fast demographic expansion of the population has taken place. Tests in the software Arlequin were carried out with 10,000 permutations.

### Genetic population structure and connectivity

The significance of population structure in *L*. *Laevigata* was tested using analysis of molecular variance (AMOVA) [46] and pairwise Φ_ST_-values. The software Arlequin (version 3.5.1.3) [40] was used to carry out both statistical calculations, applying the standard AMOVA computations, while a matrix of pairwise distances was computed with 10,000 permutations. A network of haplotypes was calculated with the programme TCS (version 1.21) [47]. Clades were defined by at least three mutational steps between each other and a star-like pattern of abundant haplotypes with connections from several singletons. Single or groups of haplotypes that were also separated by at least three or more mutational steps, but lack a star-like pattern, were not defined as clades (Fig 1D). The correlation between geographical and genetic distances among *L*. *laevigata* population was tested using a Mantel test [48], as implemented in the Isolation-by-Distance Web service IBDWS [49].

Since estimates of genetic differentiation (Φ_ST_) may be affected by unequal sample sizes, two datasets were analysed: (1) dataset excluding all sample sites with less than 10 individuals and (2) dataset with all sample sites. However, the genetic differentiation (Φ_ST_) based on the two datasets was very similar and significant. Therefore, we present the results based on all samples sites, while the results based on the reduced dataset is provided as supplementary information (S3 Table).

## Results

### Genetic diversity

Partial sequences of the mitochondrial COI gene were obtained from 138 individuals from nine sample sites in the WIO. A sequence alignment of 467 bp was obtained without indels and translated without any stop codons into an amino acid sequence. The 138 sequences yielded 61 haplotypes, of which 53 were unique to the WIO, showing 156 polymorphic sites (33%) and 184 substitutions. High values of haplotype (h) and nucleotide (π) diversity were recorded, with the average haplotype diversity and nucleotide diversity being 0.865 (0.56–1.0) and 1.9% (0.7–5.4%) respectively (Table 3). From the nine sites, two (Diani and Misali) had a haplotype diversity of 1, i.e. the sampled specimens did not share any haplotype. This could be attributed to the low sample size.

**Table 3:** Genetic diversity of the WIO population.

Sample site	Code	n	N_hp_	Genetic diversity		Neutrality tests		Mismatch distribution	
				h	π (%)	Tajima's *D*	Fu's *Fs*	SSD	HRI
Watamu	Wa	15	12	0.96	1.8	-1.42^ns^	-0.57^ns^	0.02^ns^	0.03^ns^
Mombasa	Mo	16	13	0.96	3.0	-1.48*	-1.55^ns^	0.005^ns^	0.01^ns^
Diani	Di	12	12	1.00	5.4	-0.95^ns^	-0.65^ns^	0.01^ns^	0.02^ns^
Misali, West Coast of Pemba	Mi	6	6	1.00	1.9	-1.10^ns^	-1.18^ns^	0.07^ns^	0.24^ns^
Jambiani, East Coast of Zanzibar	Ja	17	8	0.77	2.9	-1.69*	3.54^ns^	0.08^ns^	0.14^ns^
Dar es Salaam	DS	27	12	0.81	0.9	-0.56^ns^	-2.10^ns^	0.04^ns^	0.07^ns^
Mikindani	Mk	10	7	0.91	1.9	-0.83^ns^	1.87^ns^	0.05^ns^	0.12^ns^
Nosy Bé	NB	20	10	0.83	0.9	-0.17^ns^	-0.54^ns^	0.04^ns^	0.06^ns^
Tuléar	Tu	15	3	0.56	0.7	1.78^ns^	4.42^ns^	0.24*	0.46^ns^

*0.05 ≥ *P* ≥ 0.01;

ns = not significant

Values shown are: number of sequences (n), number of haplotypes (Nhp), haplotype diversity (h), nucleotide diversity (π), Tajima’s D, Fu’s Fs, sums of squares deviation (SSD), and Harpending’s raggedness index (HRI) for Linckia laevigata in the Western Indian Ocean.

### Historical demography

On the one hand, the null hypothesis of neutral evolution of the COI marker was only rejected for Mombasa and Jambiani based on Tajima's *D* test, while the results of Fu’s *Fs* test could not reject the null hypothesis for all the sites. On the other hand, the mismatch distribution analysis and Rogers’ test of sudden population expansion indicate population expansion for all sites, except Tuléar (Table 3).

### Genetic population structure and connectivity

#### Western Indian Ocean

The genetic population structure of *L*. *laevigata* in the WIO region was determined with samples collected from three sites in Kenya, four sites in Tanzania and two sites in Madagascar. AMOVA results revealed a low fixation index, but significant genetic population structure (Φ_ST_-value = 0.024, P = 0.045). After Bonferroni correction for the 36 pairwise Φ_ST_-values, the significance level was adjusted from 0.05 to 0.0014. Correspondingly, the estimated pairwise Φ_ST_-values were low and insignificant for all sites, with the exception of one site in Kenya (Diani), which was significantly differentiated from Angel reef, Tanzania (S1 Table). Three hierarchical AMOVAs were carried out based on ocean currents and geographical location (S2 Table), but only one grouping rejected the hypothesis of panmixia: (1) Kenya (Watamu, Mombasa, Diani) and Tanzanian island populations (Misali and Jambiani) and (2) the rest of the WIO sites (mainland Tanzania and Madagascar; Φ_CT_ = 0.03, P = 0.047). Nevertheless, a plot of pairwise Φ_ST_-values against geographic distance among sample sites did not exhibit a positive correlation, confirming the lack of isolation-by-distance.

#### Indo-West Pacific

Since the sequence obtained from WIO sites were shorter than the sequences reported by the two previous studies [14, 34] in the IMPA, the combined 534 sequences alignment from 36 sites was reduced to 441 bp, yielding 194 haplotypes. However, the shortening of sequences might have reduced the number of haplotypes. Evolutionary relationships of 194 *L*. *laevigata* haplotypes found in the WIO and the IMPA are presented in the haplotype network (Fig 1D), showing three different clades separated by at least three mutational steps. The highest number of shared haplotypes was seen in clade 2, which had also the most common haplotypes. All clades were characterised by a star-like structure with many singletons. The distribution of different clades across the WIO and IMPA is presented in Fig 1B and 1C. Clade 3 was restricted to the WIO, where it occured at all sites. On the one hand, clade 1 was dominant in the Visayas and Western Pacific (WP), although it appeared at lower frequencies in other sites in the central IMPA, and was completely missing in the Eastern Indian Ocean (EIO) and WIO. On the other hand, clade 2 could be found at all the sample sites in the four regions WIO, EIO, WP and central IMPA, with exception of two sites Sebakor/Sanggal/Papisol (SSP) and New Britain (NB), New Guinea.

AMOVA results for the 36 sample sites in the WIO and IMPA revealed a strong genetic structure (Φ_ST_ = 0.130, P < 0.001). The hierarchical AMOVA was also conducted for two groupings based on geographical location of sites, both rejecting the hypothesis of panmixia: (1) WIO, (2) EIO (Andaman Sea and Kupang), (3) IMPA, and (4) WP (New Britain and Biak) (Φ_CT_ = 0.199, P < 0.001), as well as (1) Indian Ocean (WIO and EIO), (2) IMPA, and (3) WP (Φ_CT_ = 0.195, P < 0.001).

## Discussion

In marine species, values less than 0.5 for both h and π (%) indicates low genetic diversity and little genetic divergence [50]. The estimated haplotype (h) and nucleotide diversities (π) in *L*. *laevigata* were higher in comparison with those shown in previous studies utilising COI in other invertebrates in the WIO, such as the giant tiger prawn *Penaeus monodon* [51], fiddler crab *Uca annulipes* [52], mangrove crab *Perisesarma guttatum* [53], but similar to the mangrove crab *Neosarmatium meinerti* [54]. The high haplotype and nucleotide diversity values might also confirm a recent population expansion that occurred with a pool of haplotypes that diversified prior to the population expansion [51]. Indeed, the results of Tajima’s *D*, Fu’s *FS*, mismatch distribution analysis, and Rogers’ tests were all consistent in indicating a sudden population expansion subsequent to a bottleneck [44–45]. This change in population size could be attributed to the reduction of habitats during the sea level low stands and recolonisation of new habitats after rising of the sea level. The sea level dropped up to 120 m in the Pleistocene, which was inevitably accompanied by loss of shelf habitats and strong fragmentation, leading to the isolation of coral reef refugia. In particular, habitat reduction was intense in areas outside the IMPA, which maintained extensive coral refugia during periods of low sea level stands [55–56]. During this time, the population of *L*. *laevigata* may have undergone dramatic decline, owing to highly reduced coral reef area. The rising temperature after the last glacial maximum was associated with sea level rise that enabled re-colonisation of shelf areas, resulting in a demographic and spatial population expansion of *L*. *laevigata* in the WIO and elsewhere. Population expansion after a population bottleneck has also been observed in the IMPA populations of *L*. *laevigata* [14, 34, 57] and in other species in the WIO, such as the anemonefish *Amphiprion akallopisos* [58], mangrove crab *Neosarmatium meinerti* [53], fiddler crab *Uca annulipes* [51], mud crab *Scylla serrata* [59], and giant tiger prawn *Penaeus monodon* [50].

Previous studies have demonstrated that genetic divergence among marine populations can occur even in the absence of any apparent barriers to dispersal [60–61]. This is also true for the WIO, where larval dispesal is greatly influenced by complex hydrographic features. Despite the long PLD of *L*. *laevigata* that provides a mechanism for long distance dispersal, AMOVA results for the population from the WIO showed a weak, but significant population structure (Φ_ST_ = 0.024, P = 0.045). This result is similar to the weak genetic structure in *L*. *laevigata* reported for populations in the IMPA, with a low fixation index (Φ_ST_ = 0.03) but significant structure [14, 34]. Shallow genetic structuring have also been observed in other marine species, such as the mud crab *Scylla serata* and the mangrove crab *Neosarmatium meinerti* along the East African coast, despite their long PLD of 3–4 weeks [54, 59]. However, the isolation-by-distance analysis was not significant in the present study, indicating that the genetic structure is not attributed to distance-restricted dispersal. Thus, reduced gene flow in WIO *L*. *laevigata* populations might be caused by local ocean currents, life history traits, topographic features or success of immigrants in mating after settlement [62]. Given that the WIO experiences complex current patterns, it is more likely that restricted gene flow in *L*. *laevigata* is caused by local oceanographic features, such as local downwelling or eddies [32]. A more pronounced, but still weak significant structure in the WIO populations was observed in the hierarchical analysis, with the following grouping: (1) Kenya (Watamu, Mombasa, and Diani) and Tanzanian Island populations (Misali and Jambiani) and (2) the rest of the WIO sites (mainland Tanzania and Madagascar). An almost identical pattern was observed in the coral *Acropora tenuis*, separating Kenyan and northern Tanzanian sites from those in the South [63]. A plausible explanation for the observed pattern of discontinuity could be due to the strong local downwelling event that occurs along the southern Kenyan and Tanzanian coast throughout the year [64–65]. This phenomenon can influence dispersal continuity, especially when planktonic larvae are transported onshore, promoting retention. Nonetheless, the population structure revealed by hierarchical AMOVA analyses was weak, suggesting that substantial gene flow occurs due to their long PLD, even in the presence of local downwelling.

Numerous studies have shown a sharp genetic break between Indian and Pacific Ocean *L*. *laevigata* populations due to sea level fluctuations in the Pleistocene [9, 14, 21, 33, 34]. However, these previous studies could not provide a full phylogeographic context of *L*. *laevigata*, because their sampling efforts were concentrated in the IMPA. The present study complements these previous investigations by combining the sequence data from the WIO and IMPA. The AMOVA for the combined data sets revealed a strong genetic structure (Φ_ST_ = 0.13, P < 0.001), comparable with other studies on *L*. *laevigata* in the Indo-West Pacific [21, 33]. Moreover, the strong genetic differentiation in the Indo-West Pacific has also been shown in other species, such as the giant tiger prawn *Penaeus monodon* [50, 66], the crown-of-thorn sea star *Acanthaster planci* [67], and the holothurians *Holothuria atra* and *Holothuria nobilis* [68–69]. The genetic differentiation between the Indian and Pacific Ocean populations in *L*. *laevigata* is consistent with limited gene flow between the two ocean basins for tens of thousands of years [33, 67]. The Torres Strait between Australia and New Guinea was closed throughout the Pleistocene, while the Indonesian throughflow might have been restricted [70]. It is unlikely that dispersal in *L*. *laevigata* could have happened either in the past or present along the southeastern coast of Australia because the larvae of this tropical species would not survive the low temperate temperatures [33]. Furthermore, upwelling of cold water at the base of the Indonesian arc blocked the movement of tropical marine larvae through the few opened narrow channels in the eastern Indonesian islands, effectively closing any dispersal routes between the Pacific and Indian Ocean [33]. Conversely, a number of species demonstrate a lack of this apparent phylogeographic break. These include the marine snail *Echinolittorina reticulata* [13], the soldierfish *Myripristis berndi* [71], the swordfish *Xiphius gladius*, and the mud crab *Scylla serrata* [72]. This lack of an apparent phylogeographic break is attributed to the re-establishment of dispersal rapidly after glacial maxima. Hierarchical analysis displayed a stronger genetic break with the following groupings: (1) WIO, (2) EIO, (3) IMPA and (4) WP, as well as (1) Indian Ocean (WIO and EIO), (2) IMPA and (3) WP. The first hierarchical grouping shows that the WIO populations are genetically distinct from the EIO populations, suggesting a WIO-EIO divide. This break within the Indian Ocean was also observed in the giant clams *Tridacna maxima* and *Tridacna squamosa* [73], the anemonefish *Amphiprion akallopisos* [58], the crown-of-thorns sea star *Acanthaster planci* [74], and the black tiger prawn *Penaeus monodon* [50, 66]. The limited genetic exchange between the WIO and EIO supports the notion that fewer islands exist in the Indian Ocean compared to the West Pacific in order to facilitate dispersal through “island hopping”. Moreover, the enclosure of the Andaman Sea by land following sea level regression during the Pleistocene isolated the sample site in Thailand (AS) partially from the Indian Ocean and completely from the Pacific Ocean.

The genetic break between the Indian and Pacific Ocean is also corroborated by the geographical distribution of the different clades (Fig 1). On the one hand, clade 1 predominates in the WP and is completely missing in the EIO and WIO. On the other hand, clade 2 is found in almost all sites, with the exception of Sebakor/Sanggal/Papisol (SSP) and New Britain (NB), New Guinea. Clade 2 occurs in the WIO and was the only clade sampled in the two EIO sites (Andaman Sea (AS) and Kupang (Ku)). It also dominated the sites in the central IMPA, an indication that it is likely the ancestral Indian Ocean clade. Clade 1 most probably evolved in the Pacific Ocean and its westward dispersal into the central IMPA by the ITF is reduced by the Mindanao and Halmahera Eddies. Mixing of clades 1 and 2 occurs in the central IMPA, despite the genetic barrier that is observed in many species between the Indian and Pacific Ocean. Clade 3 has evolved and accumulated in the WIO and its restriction to this region also indicates limited gene flow between populations in the WIO and their counterparts in the EIO, IMPA, and WP. Clades 2 and 3 were found at all sample sites in the WIO, which could be attributed to the bifurcation of the South Equatorial Current, forming the north-flowing East African Coast Current and the south-flowing Mozambique Current. These currents might be responsible for the wide distribution of clades 2 and 3 throughout the WIO.

## Supporting Information

S1 Table Pairwise Φ_ST_-values among populations of *Linckia laevigata* in the Western Indian Ocean (for abbreviations see Table 1). (DOCX)

S2 Table Hierarchical AMOVA based on mitochondrial control region sequences from *Linckia laevigata* with alternative groupings of sample sites from the WIO. (DOCX)

S3 Table AMOVA results on dataset that excludes all sample sites with less than 10 individuals. (DOCX)

## References

[1] Kochzius M, Nuryanto A. Strong genetic population structure in the boring giant clam, *Tridacna crocea*, across the Indo-Malay Archipelago: implications related to evolutionary processes and connectivity. Mol ecol. 2008; 17:3775–3787. 10.1111/j.1365-294X.2008.03803.x 18662232

[2] Mora C, Andrèfouët S, Costello M J, Kranenburg C, Rollo A, Veron J, et al Ecology. Coral reefs and the global network of Marine Protected Areas. Science. 2006; 312:1750–1751. 10.1126/science.1125295 16794065

[3] Hubert N, Meyer C, Bruggemann JH, Guérin F, Komeno RJL, Espiau B, et al Cryptic diversity in Indo-Pacific coral reef fishes revealed by DNA-barcoding provides new support to the centre-of-overlap hypothesis. PLoS One. 2012; 7: e28987 10.1371/journal.pone.0028987 22438862 PMC3305298

[4] Gaither MR, Rocha LA. Origins of species richness in the Indo-Malay-Philippine biodiversity hotspot: evidence for the centre of overlap hypothesis. J Biogeogr. 2013; 40:1638–1648.

[5] Voris HK. Maps of Pleistocene sea levels in Southeast Asia: shorelines, river systems and time durations. J Biogeogr. 2000; 27:1153–1167.

[6] Timm J, Figiel M, Kochzius M. Contrasting patterns in species boundaries and evolution of anemonefishes (Amphiprioninae, Pomacentridae) in the centre of marine biodiversity. Mol Phylogenet Evol. 2008; 49: 268–276. 10.1016/j.ympev.2008.04.024 18513996

[7] Kochzius M, Söller R, Khalaf MA, Blohm D. Molecular phylogeny of the lionfish genera *Dendrochirus* and *Pterois* (Scorpaenidae, Pteroinae) based on mitochondrial DNA sequences. Mol Phylogenet Evol. 2003; 28: 396–403. 12927126 10.1016/s1055-7903(02)00444-x

[8] McMillan WO, Palumbi SR. Concordant evolutionary patterns among Indo-West Pacific butterflyfishes. Proc R Soc B Biol Sci. 1995; 260:229–236. 10.1098/rspb.1995.0085 7784441

[9] Williams ST. (2000). Species boundaries in the starfish genus *Linckia*. Mar Biol 136:1137–148.

[10] Lessios HA, Kessing BD, Robertson DR, Paulay G. Phylogeography of the pantropical sea urchin *Eucidaris* in relation to land barriers and ocean currents. Evolution. 1999; 53: 806–817. 28565646 10.1111/j.1558-5646.1999.tb05374.x

[11] Lessios HA, Kessing BD, Pearse JS. Population structure and speciation in tropical seas: global phylogeography of the sea urchin *Diadema*. Evolution. 2001; 55:955–975. 11430656 10.1554/0014-3820(2001)055[0955:psasit]2.0.co;2

[12] Lessios HA, Kane J, Robertson DR. Phylogeography of the pantropical sea urchin *Tripneustes*: contrasting patterns of population structure between oceans. Evolution. 2003; 57:2026–2036. 14575324 10.1111/j.0014-3820.2003.tb00382.x

[13] Reid DG, Lal K, Mackenzie-Dodds J, Kaligis F, Littlewood DTJ, Williams ST. Comparative phylogeography and species boundaries in *Echinolittorina* snails in the central Indo-West Pacific. J Biogeogr. 2006; 33:990–1006.

[14] Kochzius M, Seidel C, Hauschild J, Kirchhoff S, Mester P, Meyer-Wachsmuth I, et al Genetic population structures of the blue starfish *Linckia laevigata* and its gastropod ectoparasite *Thyca crystallina*. Mar Ecol Prog Ser. 2009; 396:211–219.

[15] Vogler C, Benzie JH, Lessios H, Barber P, Wörheide G. A threat to coral reefs multiplied? Four species of crown-of-thorns starfish. Biol Lett. 2008; 4:696–699. 10.1098/rsbl.2008.0454 18832058 PMC2614177

[16] Hellberg ME, Burton RS, Neigel JE, Palumbi SR. Genetic assessment of connectivity among marine populations. Bull Mar Sci. 2002; 70:273–290.

[17] Imron, Jeffrey B, Hale P, Degnan B M, Degnan SM. Pleistocene isolation and recent gene flow in *Haliotis asinina*, an Indo-Pacific vetigastropod with limited dispersal capacity. Mol Ecol. 2007; 16:289–304. 10.1111/j.1365-294X.2006.03141.x 17217345

[18] Ayre DJ, Hughes TP. Genotypic diversity and gene flow in brooding and spawning corals along the Great Barrier Reef, Australia. Evolution. 2000; 54:1590–1605. 11108587 10.1111/j.0014-3820.2000.tb00704.x

[19] Barber PH, Palumbi SR, Erdmann MV, Moosa M. Sharp genetic breaks among populations of *Haptosquilla pulchella* (Stomatopoda) indicate limits to larval transport: patterns, causes, and consequences. Mol Ecol. 2002; 11:659–674. 11972755 10.1046/j.1365-294x.2002.01468.x

[20] York KL, Blacket MJ, Appleton BR. The Bassian Isthmus and the major ocean currents of southeast Australia influence the phylogeography and population structure of a southern Australian intertidal barnacle *Catomerus polymerus* (Darwin). Mol Ecol. 2008; 17:1948–1961. 10.1111/j.1365-294X.2008.03735.x 18363669

[21] Crandall ED, Treml EA, Liggins L, Gleeson L, Yasuda N, Barber PH, et al Return of the ghosts of dispersal past: historical spread and contemporary gene flow in the blue sea star *Linckia laevigata*. Bull Mar Sci. 2014; 90: 399–425.

[22] Clark AM, Rowe FEW. Shallow-water Indo-West-Pacific echinoderms. Trustees of the British museum (natural history). 1971; pp. 238.

[23] Yamaguchi M. Population structure, spawning, and growth of the coral reef asteroid *Linckia laevigata* (Linnaeus). Pac Sci. 1977; 31:13–30.

[24] Williams ST, Benzie JH. Genetic consequences of long larval life in the starfish *Linckia laevigata* (Echinodermata: Asteroidea) on the Great Barrier Reef. Mar Biol. 1993; 117:71–77.

[25] Yamaguchi M. Early life histories of coral reef asteroids, with special reference to *Acanthaster planci* (L.) In Jones OA, Endean R (eds) Biology and geology of coral reefs, Academic Press, New York and London; 1973 pp. 369–387

[26] Sheppard CRC. Coral reefs of the Western Indian Ocean: An overview. In: McClanahan T.R., Sheppard C.R.C. & Obura D.O. Oxford University Press, New york; 2000 pp. 3–38.

[27] Muths D, Tessier E, Bourjea J. Genetic structure of the reef grouper *Epinephelus merra* in the Western Indian Ocean appears congruent with biogeographic and oceanographic boundaries. Mar Ecol. 2014; 36:447–461.

[28] Wyrtki K. Physical Oceanography of the Southeast Asian Waters. University of California La Jolla, California; 1961.

[29] Gordon AL, Fine RA. Pathways of water between the Pacific and Indian oceans in the Indonesian seas. Nature. 1996; 379:146–149.

[30] Gordon AL. (2005). Oceanography of the Indonesian seas and their throughflow. Oceanography. 2005; 18:14–27.

[31] Schouten MW, de Ruijter WPM, van Leeuwen PJ, Ridderinkhof H. Eddies and variability in the Mozambique Channel. Deep-Sea Res II. 2003; 50:1987–2003.

[32] Schott FA, McCreary JP. The monsoon circulation of the Indian Ocean. Prog Oceanogr. 2001; 51:1–123

[33] Williams ST, Benzie JH. Evidence of a biogeographic break between populations of a high dispersal starfish: congruent regions within the Indo-West Pacific defined by color morphs, mtDNA and allozyme data. Evolution. 1998; 52:87–99. 28568135 10.1111/j.1558-5646.1998.tb05141.x

[34] Alcazar SD, Kochzius M. Genetic population structure of the blue sea star *Linckia laevigata* in the Visayas (Philippines). J Mar Biol Assoc UK. 2016; 96: 707–713

[35] Folmer O, Black M, Hoeh W, Lutz R, Vrijenhoek R. DNA primers for amplification of mitochondrial cytochrome c oxidase subunit I from diverse metazoan invertebrates. Mol Mar Biol Biotechnol. 1994; 3:294–299. 7881515

[36] Thompson JD, Higgins DG, Gibson TJ. CLUSTAL W: improving the sensitivity of progressive multiple sequence alignment through sequence weighting, position-specific gap penalties and weight matrix choice. Nucleic Acids Res. 1994; 22:4673–4680. 7984417 10.1093/nar/22.22.4673 PMC308517

[37] Hall TA. BioEdit: a user-friendly biological sequence alignment editor and analysis program for Windows 95/98/NT. Nucleic Acids Symp Ser. 1999; 41:95–98.

[38] Nei M. Molecular evolutionary genetics. Columbia University Press, New York; 1987.

[39] Nei M, Jin L. Variances of the average numbers of nucleotide substitutions within and between populations. Mol Biol Evo. 1989; l 6:290–300. 10.1093/oxfordjournals.molbev.a040547 2576093

[40] Excoffier L, Lischer HEL. Arlequin suite version 3.5: a new series of programs to perform population genetics analyses under Linux and Windows. Mol Ecol Resour. 2010; 10:564–567. 10.1111/j.1755-0998.2010.02847.x 21565059

[41] Tajima F. Statistical method for testing the neutral mutation hypothesis by DNA polymorphism. Genetics. 1989; 123:585–595. 2513255 10.1093/genetics/123.3.585 PMC1203831

[42] Fu YX. Statistical Tests of Neutrality of Mutations against Population Growth, Hitchhiking and Background Selection. Genetics. 1997; 147:915–925. 9335623 10.1093/genetics/147.2.915 PMC1208208

[43] Schneider S, Excoffier L. Estimation of past demographic parameters from the distribution of pairwise differences when the mutation rates vary among sites: application to human mitochondrial DNA. Genetics. 1999; 152:1079–1089. 10388826 10.1093/genetics/152.3.1079 PMC1460660

[44] Rogers AR, Harpending H. Population-Growth Makes Waves In The Distribution Of Pairwise Genetic-Differences. Mol Biol and Evol. 1992; 9:552–569. 1316531 10.1093/oxfordjournals.molbev.a040727

[45] Rogers AR. Genetic evidence for a Pleistocene population explosion. Evolution. 1995; 49:608–615. 28565146 10.1111/j.1558-5646.1995.tb02297.x

[46] Excoffier L, Smouse PE, Quattro JM. Analysis of Molecular Variance Inferred from Metric Distances among DNA Haplotypes: Application to Human Mitochondrial DNA Restriction Data. Genetics. 1992; 131:479–491. 1644282 10.1093/genetics/131.2.479 PMC1205020

[47] Clement M, Posada D, Crandall KA. TCS: a computer program to estimate gene genealogies. Mol Ecol. 2000; 9:1657–1659. 11050560 10.1046/j.1365-294x.2000.01020.x

[48] Mantel N. The detection of disease clustering and a generalized regression approach. Cancer Res. 1967; 27:209–220. 6018555

[49] Jensen JL, Bohonak AJ, Kelley ST. Isolation by distance, web service. BMC Genetics. 2005; 6:1–6. 15760479 10.1186/1471-2156-6-13 PMC1079815

[50] Grant WS, Bowen BW. Shallow population histories in deep evolutionary lineages of marine fishes: insights from sardines and anchovies and lessons for conservation. J Hered. 1998; 89:415–426.

[51] Benzie JH, Ballment E, Forbes T, Demetriades NT, Sugama K, Haryanti, et al Mitochondrial DNA variation in Indo-Pacific populations of the giant tiger prawn, *Penaeus monodon*. Mol Ecol. 2002; 11:2553–2569. 12453239 10.1046/j.1365-294x.2002.01638.x

[52] Silva IC, Mesquita N, Paula J. Lack of population structure in the fiddler crab *Uca annulipe*s along an East African latitudinal gradient: genetic and morphometric evidence. Mar Biol. 2010; 157:1113–1126.

[53] Silva IC, Mesquita N, Paula J. Genetic and morphological differentiation of the mangrove crab *Perisesarma guttatum* (Brachyura: Sesarmidae) along an East African latitudinal gradient. Biol J Linn Soc. 2010; 99: 28–46.

[54] Ragionieri L, Cannicci S, Schubart CD, Fratini S. Gene flow and demographic history of the mangrove crab *Neosarmatium meinerti*: A case study from the western Indian Ocean. Estuar Coast Shelf Sci. 2010; 86:179–188.

[55] Hoareau TB, Boissin E, Paulay G, Bruggemann JH. The Southwestern Indian Ocean as a potentiel marine evolutionary hostpot: perspectives from compararive phylogeography of reef brittle-stars. J Biogeogr. 2013; 40:2167–2179.

[56] Pellissier L, Leprieur F, Parravicini V, Cowman PF, Kulbicki M, Litsios G, et al Quaternary coral reef refugia preserved fish diversity. Science. 2014; 344:1015–1019. 10.1126/science.1249853 24876495

[57] Crandall ED, Jones ME, Muñoz MM, Akinronbi B, Erdmann MV, Barber PH. Comparative phylogeography of two seastars and their ectosymbionts within the Coral Triangle. Mol Ecol. 2008; 17:5276–5290. 10.1111/j.1365-294X.2008.03995.x 19067797

[58] Huyghe F, Kochzius M. Highly restricted gene flow between disjunct populations of the skunk clownfish (*Amphiprion akallopisos*) in the Indian Ocean. Mar Ecol. 2016;

[59] Fratini S, Vannini M. Genetic differentiation in the mud crab *Scylla serrata* (Decapoda: Portunidae) within the Indian Ocean. J Experi Mar Biol Ecol. 2002; 272:103–116.

[60] Fauvelot C, Planes S. Understanding origins of present-day genetic structure in marine fish: biologically or historically driven patterns? Mar Biol. 2002; 141:773–788.

[61] Taylor MS, Hellberg ME. Marine radiations at small geographic scales: speciation in neotropical reef gobies (*Elacatinus*). Evolution. 2005; 59:374–385. 15807422

[62] Burton RS, Feldman MW. Population genetics of coastal and estuarine invertebrates: does larval behavior influence population structure? In Kennedy V. S. (Ed.) Estuarine Comparisons Academic Press; 1982 pp. 537–551.

[63] van der Ven RM, Triest L, De Ryck DJR, Mwaura JM, Mohammed MS, Kochzius M. Population genetic structure of the stony coral *Acropora tenuis* shows high but variable connectivity in East Africa. J Biogeogr. 2015; 10.1111/jbi.12643

[64] Bell BE. Marine fisheries In: Morgan W. T. W. (ed.) East Africa: its people and resources. Oxford University Press, London; 1972 pp. 243–254.

[65] McClanahan TR. Seasonality in East Africa’s coastal waters. Mar Ecol Prog Ser. 1988; 44:191–199.

[66] Duda TF, Palumbi SR. Population structure of the black tiger prawn, *Penaeus monodon*, among western Indian Ocean and western Pacific populations. Mar Biol. 1999; 134:705–710.

[67] Benzie JH. Major genetic differences between crown-of-thorns starfish (*Acanthaster planci*) populations from the Indian and Pacific Oceans. Evolution. 1999; 53:1782–1795. 28565442 10.1111/j.1558-5646.1999.tb04562.x

[68] Uthicke S, Benzie JH. Restricted gene flow between *Holothuria scabra* (Echinodermata: Holothuroidea) populations along the north-east coast of Australia and the Solomon Islands. Mar Ecol Prog Ser. 2001; 216:109–117.

[69] Uthicke S, Benzie JH. Gene flow and population history in high dispersal marine invertebrates: mitochondrial DNA analysis of *Holothuria nobilis* (Echinodermata: Holothuroidea) populations from the Indo-Pacific. Mol Ecol. 2003; 12:2635–2648. 12969467 10.1046/j.1365-294x.2003.01954.x

[70] Galloway RW, Kemp EM. “Late Cainozoic environments in Australia.” Ecological Biogeography of Australia, Keast A (ed.); 1981. pp. 51–80.

[71] Craig MT, Eble JA, Bowen BW, Robertson DR. High genetic connectivity across the Indian and Pacific Oceans in the reef fish *Myripristis berndti* (Holocentridae). Mar Ecol Prog Ser. 2007; 334:245–254.

[72] Gopurenko D, Hughes JM, Keenan CP. Mitochondrial DNA evidence for rapid colonisation of the Indo-West Pacific by the mudcrab *Scylla serrata*. Mar Biol. 1999; 134:227–233.

[73] Hui M, Kraemer WE, Seidel C, Nuryanto A, Joshi A, Kochzius M. Comparative genetic population structure of three endangered giant clams (Cardiidae: *Tridacna species*) throughout the Indo-West Pacific: implications for divergence, connectivity and conservation. J Mollus Stud. 2016; 10.1093/mollus/eyw001

[74] Vogler C, Benzie JH, Barber P, Erdmann M, Ambariyanto, Sheppard C, et al Phylogeography of the crown-of-thorns starfish in the Indian Ocean. PLoS ONE. 2012; 7:e43499 10.1371/journal.pone.0043499 22927975 PMC3424128

