# Supplementary material for: Genetic Population Structure of the Coral Reef Sea Star Linckia laevigata in the Western Indian Ocean and Indo-West Pacific
Source: PLoS One. 2016 Oct 31;11(10):e0165552. doi: 10.1371/journal.pone.0165552 (PMC5087890; doi:10.1371/journal.pone.0165552)
Supplement: S1 Table — (DOCX) [file pone.0165552.s001.docx]

S1Table. Pairwise Φ_ST_-values among populations of *Linckia laevigata* in the Western Indian Ocean (for abbreviations see Table 1).

|  | **NB** | **Tu** | **Mo** | **Wa** | **Di** | **DS** | **Ja** | **Mi** |
| --- | --- | --- | --- | --- | --- | --- | --- | --- |
| **Tu** | -0.0486^ns^ |  |  |  |  |  |  |  |
| **Mo** | 0.0101^ns^ | 0.0045^ns^ |  |  |  |  |  |  |
| **Wa** | -0.0204^ns^ | -0.0171^ns^ | -0.0304^ns^ |  |  |  |  |  |
| **Di** | 0.1221^ns^ | 0.1113^ns^ | 0.0009^ns^ | 0.0366^ns^ |  |  |  |  |
| **DS** | -0.0096^ns^ | 0.0114^ns^ | 0.0467^ns^ | -0.0014^ns^ | 0.1519* |  |  |  |
| **Ja** | 0.0044^ns^ | -0.0058^ns^ | -0.0074^ns^ | -0.0087^ns^ | 0.0582^ns^ | 0.0401^ns^ |  |  |
| **Mi** | 0.0622^ns^ | 0.0626^ns^ | -0.0474^ns^ | -0.0132^ns^ | -0.0072^ns^ | 0.141^ns^ | -0.0323^ns^ |  |
| **Mk** | 0.0004^ns^ | -0.0014^ns^ | 0.0228^ns^ | -0.0238^ns^ | 0.0333^ns^ | 0.0231^ns^ | -0.0088^ns^ | -0.013^ns^ |

**P* ≤0.001; ns = not significant
