# Supplementary material for: Genetic Population Structure of the Coral Reef Sea Star Linckia laevigata in the Western Indian Ocean and Indo-West Pacific
Source: PLoS One. 2016 Oct 31;11(10):e0165552. doi: 10.1371/journal.pone.0165552 (PMC5087890; doi:10.1371/journal.pone.0165552)
Supplement: S2 Table — (DOCX) [file pone.0165552.s002.docx]

S2 Table. Hierarchical AMOVA based on mitochondrial control region sequences from *Linckia laevigata* with alternative groupings of sample sites from the WIO.

| **Grouping** | **Φ_CT_** | **P value** |  |
| --- | --- | --- | --- |
| **WIO** |  |  |  |
| **(Wa,Mo,Di,Ja,Mi)(DS,Mk,Nb,Tu)** | **0.03465** | **0.04718** |  |
| (Nb, Tu) (Wa, Mo, Di, Ja, DS, Mi, Mk) | -0.005 | 0.44385 |  |
| (Nb, Tu,MK) (Wa, Mo, Di, Ja, DS, Mi) | -0.0018 | 0.35862 |  |
|  |  |  |  |
|  |  |  |  |
|  |  |  |  |

*0.05 ≥ *P* ≥ 0.01; **0.01 > *P* ≥ 0.001; ****P* < 0.001; ns= not significant.
