# Supplementary material for: Genetic Population Structure of the Coral Reef Sea Star Linckia laevigata in the Western Indian Ocean and Indo-West Pacific
Source: PLoS One. 2016 Oct 31;11(10):e0165552. doi: 10.1371/journal.pone.0165552 (PMC5087890; doi:10.1371/journal.pone.0165552)
Supplement: S3 Table — (DOCX) [file pone.0165552.s003.docx]

S3Table. AMOVA results on dataset that excludes all sample sites with less than 10 individuals

| **AMOVA** | **Φ_ST_** | **P value** |  |
| --- | --- | --- | --- |
| WIO | 0.025 | 0.02 |  |
| Indo-West Pacific | 0.17 | P < 0.001 |  |
|  |  |  |  |
|  |  |  |  |
|  |  |  |  |

*0.05 ≥ *P* ≥ 0.01; **0.01 > *P* ≥ 0.001; ****P* < 0.001; ns= not significant.
